# Prevalence of caregiver burden, depressive and anxiety symptoms in caregivers of children with psychiatric disorders in Durban, South Africa

**DOI:** 10.4102/sajpsychiatry.v24i0.1314

**Published:** 2018-09-20

**Authors:** Saeeda Paruk, Mayuri Ramdhial

**Affiliations:** 1 Department of Psychiatry, University of Kwazulu-Natal, South Africa

## Abstract

**Background:**

There is increased caregiver burden, depressive and anxiety symptoms associated with the care of mentally ill children. This may be influenced by child or caregiver factors such as socio-demographic and clinical factors and has not been explored in the South African context.

**Aim:**

To describe the prevalence of depression, anxiety symptoms and caregiver burden in caregivers of children treated at psychiatric outpatient services at two public sector hospitals.

**Methods:**

A cross-sectional questionnaire study of 121 adult primary caregivers of children aged 1–17 years with mental illness using a socio-demographic questionnaire, Patient Health Questionnaire (PHQ-9), Generalised Anxiety Disorder-7 Questionnaire (GAD-7), and the Child and Adolescent Impact Assessment (CAIA) to assess caregiver burden.

**Results:**

The caregivers were predominantly female (*n* = 96, 79.5%) and married (*n* = 72, 59.5%), with a mean age of¬34.99 years (SD 10.38), and 74% were mothers. Among the children, there was a predominance of boys with a 1:4 ratio of girls to boys. The most common diagnoses in the children were attention deficit hyperactivity disorder (ADHD) (*n* = 56, 59.6%) and autism spectrum disorder (*n* = 22, 23.4%). Fifty-four (44%) caregivers were depressed with a mean PHQ9 score of 5.75 (SD 5.98), and 65 (54 %) reported anxiety symptoms with a mean GAD7 score of 5.71 (SD 5.03). Mothers reported significantly higher levels of anxiety (*p* = 0.045) and experienced higher impact on feelings of personal well-being on the CAIA (*p* = 0.004) in comparison with fathers. Caregiver burden was predominantly reported in the domains of restrictions in activities (*n* = 40, 32.8%), feelings of personal well-being (*n* = 37, 30.7%) and economic impact (*n* = 21, 17.4%).

The caregivers of children with ADHD reported higher anxiety levels (*p* = 0.023) than for autistic children. A diagnosis of autistic spectrum disorder was associated with higher income impact (*p* = 0.004) and restrictions impact (*p* = 0.001) than for children with ADHD diagnosis in terms of caregiver burden.

**Conclusion:**

The high prevalence of depression and anxiety symptoms reported amongst caregivers suggests the need for improved mental health screening and psycho-social support programmes for caregivers, particularly mothers. Programmes should consider the impact of caregiving, particularly on mental health, income and social restrictions of caregivers.

